# Effectiveness of sub-albumin protein leakage membrane EMIC2 in post-cardiac surgery rhabdomyolysis

**DOI:** 10.1186/cc13587

**Published:** 2014-03-17

**Authors:** G Paternoster, A Covino, R Mercorella, C Di Leo, M Calabrese, G Pittella

**Affiliations:** 1 San Carlo Hospital, Potenza, Italy

## Introduction

A high postoperative serum myoglobin (MyG) concentration predicts the incidence of acute kidney injury (AKI) and need for renal replacement therapy (RRT), as reported in several surgical settings. The incidence of such events in the ICU is reported to be 2 to 5% of all causes of AKI [1], but can even worsen in the case of cardiac surgery (40.3%) [1]. MyG is a small protein (17.8 kDa) that can be removed with RRT, typically in convection cases. New-generation membranes, removing sub-albumin protein molecular weight solutes, can be used in diffusive treatments (CVVHD) with the advantage of limiting albumin loss and easily combining with citrate anticoagulation, pivotal for cardio-surgical settings. We assessed the effectiveness of EMIC2 with citrate anticoagulation in AKI prevention of post-cardiac surgical patients.

## Methods

This is a case series of eight patients (mean age 62.7 years, five male, EuroSCORE log 15.61) in cardiac surgery on CPBP for 150 minutes and mean aortic cross-clamping of 98 minutes (range 25 to 190). We measured MyG, procalcitonin (PCT), and creatinin (sCr) at ICU admission and, if serum MyG was higher than 600 mg/dl, the patient was treated with CVVHD-EMIC2-citrate anticoagulant within 12 hours of ICU admission for 72 hours and a dose of 2,000 ml/hour. Biochemical assays were obtained at 12, 24, and 72 hours and at ICU discharge.

## Results

**Figure 1:**
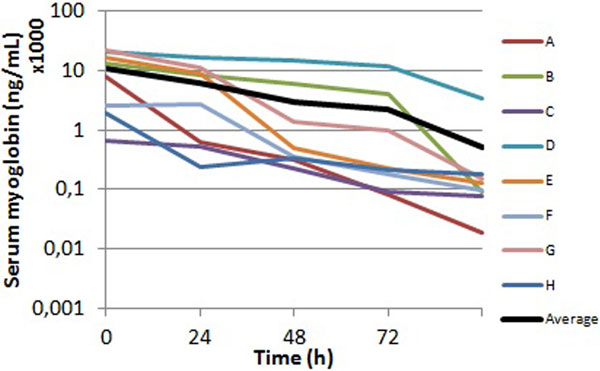


The pretreatment MyG median value was 10,789 ng/ml; it significantly reduced on average 92.8% during CVVHD (see Figure 1) and it remained low at ICU discharge (median value 114 ng/ml). sCr remained stable (average time value equal to 0.94 mg/dl) during CVVHD; PCT also decreased over time with a reduction rate equal to 78% (from 5.35 ± 4.39 mg/dl to 1.23 ± 1.09 mg/dl at the end of CVVHD). Finally, six patients survived at 90 days.

## Conclusion

This small experience confirms that serum MyG is likely to increase in post-cardiac surgical high-risk patients and suggests a beneficial effect of CRRT treatments with EMIC2 membranes and citrate on serum MyG, potentially preventing AKI. Further larger assessment can be advised for confirmation.
